# Morphology and Crystallinity of Urea-Formaldehyde Resin Adhesives with Different Molar Ratios

**DOI:** 10.3390/polym13050673

**Published:** 2021-02-24

**Authors:** Ji Li, Yifu Zhang

**Affiliations:** School of Resources, Environment and Materials, Guangxi University, Nanning 530004, China; jasonlee0803@163.com

**Keywords:** urea-formaldehyde resin, electron microscope, X-ray diffraction, crystallinity, curing agent

## Abstract

Using formaldehyde and urea as raw materials, a stable urea–formaldehyde resin (UF) is synthesized by the “alkali-acid-alkali” method. Unlike most thermosetting resins, UF often shows the appearance of crystal domains. In order to understand the relationship between the crystal and morphology of UF resin, analysis was carried out with the help of polarizing microscopy (POM), scanning electron microscopy (SEM), X-ray diffraction (XRD), transmission electron microscopy (TEM) and Fourier transform infrared spectroscopy (FT-IR). The changes of two kinds of UF resins with molar ratios (F/U) of 1.4 and 1.0 before and after curing and under the influence of different curing agents and additives were studied. SEM results showed that the UF resins with low F/U (1.0) show spherical or flat structures before and after curing, and the diameter of the spherical structure increases with the increase of the content of curing agent, while in the UF resin with high F/U (1.4) it is difficult to observe the above phenomenon. At the same time, the possible accumulation mode of UF colloidal particles in the process of aggregation is explained, and the curing agent obviously promotes the development of the crystal structure, which may be the reason for the emergence of a large number of spherical particles. XRD results showed that the resin with low F/U has higher crystallinity than the resin with high F/U, indicating that the former shows more crystallization regions, while the latter shows more amorphous structure, and the crystallinity increases with the increase of the curing agent content, but the position of the crystallization peak does not change with the type of curing agent and the amount of curing agent. Observation of the selected area electron diffraction (SAED) pattern obtained by TEM shows that the cured low F/U (1.0) resin has a polycrystalline structure and a body-centered cubic unit cell. FT-IR results showed that the linear segment, branched structure, hydroxymethyl and methylene structure changes in UF affect the formation of crystal structure. This study also shows the possible contribution of hydroxymethylated species to the formation of crystals.

## 1. Introduction

Urea-formaldehyde (UF) resin is the most widely used adhesive in the wood industry. It is a water-based polymer resin produced by urea and formaldehyde under the catalysis of acid or alkali [1,2]; UF resin has a wide range of raw materials and low cost, high strength, fast curing and other advantages. However, the release of formaldehyde in the production and use of UF resin and its products, and the deterioration of durability under high temperature or high humidity environments, have greatly affected the application of UF resin in the wood industry [3,4,5]. In addition, formaldehyde release from indoor panels is one of the main factors leading to sick building syndrome in indoor environment; therefore, formaldehyde release has always been one of the most important aspects of UF resin research [6,7,8]. In industrial production, the commonly used methods to reduce the release of formaldehyde mainly include reducing the molar ratio F/U of formaldehyde to urea; strictly controlling the reaction process conditions of UF resin synthesis, such as pH value, reaction temperature and reaction time; using formaldehyde traps, such as urea, polyvinyl alcohol, melamine; among these methods, the most effective is to reduce the molar ratio (F/U) [9,10,11]. In the current industrial production, in order to reduce formaldehyde emissions, the molar ratio of formaldehyde to urea is generally controlled between 0.9 and 1.0 [3,11].

It is well known that the UF resin with low molar ratio F/U has lower formaldehyde emission, but it is often at the expense of bonding strength [12,13]. Some studies have shown that the crystal structure of low molar ratio UF resin is the main reason for poor adhesion [13,14,15]. Myers [16] found that the free formaldehyde in UF resin and the hydrolysis of UF resin in acidic and humid environment are the reasons for formaldehyde release from wood-based panels. Park [17] found that the hydrolysis of cured UF resin is related to its chemical structure and cross-linking degree, and its crystallization region helps to improve the hydrolytic stability. Pratt [18] studied the colloidal particles of UF resin in detail, found that the crystal structure was related to the colloidal particles, and considered that the protonated formaldehyde around the colloidal particles played a protective role on the colloids. Motter [19] found that the colloidal particles are composed of 4–8 units of urea, depending on the molar ratio F/U. Stuligross [14] studied the colloid properties and crystallinity of UF resin, which showed that the resin formula would not change the crystal structure, but only the percentage of crystallinity. Dunker [20,21] et al. found that the UF resin contains semi-crystalline colloidal regions, and the physical association in the resin solution is related to the solid crystalline regions, and the high order of the crystal structure is attributed to the hydrogen bonding between the colloidal particles. Wibowo [22] studied in detail the effect of hydrogen bond on the crystallization of UF resin and found that in the stage of adding the last batch of urea, the hydrogen bond between the linear molecules obtained by the cleavage of formaldehyde and methylene ether bond of branched chain formed the crystal domain of UF resin.

By means of molecular dynamics simulation and experiment, Pizzi [23] found that the main species adhering to cellulose were hydroxymethylated oligomers, and the lower molar ratio of F/U would reduce the utilization rate of hydroxymethylated substances. Dazmiri [24] studied the effect of initial molar ratio on the adhesion properties of UF resin products, and found that the UF resin with the highest proportion of linear hydroxymethyl groups in the initial stage had the best adhesion properties, while the UF resin with the highest proportion of methylene bonds in the initial stage had the worst adhesion properties and the lowest formaldehyde emission. The properties of cured UF resin are closely related to the properties of finished UF resin and formaldehyde emission [9,10,25,26]. Therefore, the research on the properties and formation mechanism of resin cured materials has become a problem of close attention. Ferg [27] found that the crystalline or orderly stacked structure in UF resin does not promote the formation of a three-dimensional network structure on the bonding line. Park [7,13,28] et al. did a lot of research on the crystal structure of UF resin, he found that even if the UF resin cured in the presence of wood, the tracheal cavity of the wood still contains various forms of polycrystalline. When the crystals in UF resin are inhomogeneously dispersed, it may cause the adhesion performance of the UF resin to decrease [23].

Some studies have shown that the crystal region of cured UF resin is mainly affected by molar ratio F/U and curing agent, and different types of curing agent directly affect the curing of UF resin, and the formaldehyde release, bonding strength and other properties also show great differences [25,29,30,31]. At the same time, the formation of UF resin is often accompanied by changes in the morphology and crystallinity of the resin. Understanding the changes of these microscopic information and obtaining more convincing evidence will help the research on the curing mechanism of UF resin. Therefore, it is necessary to know more about the crystal structure and formation mode of UF resin.

In the past 40 years, the research on UF resin has mostly focused on reducing formaldehyde emissions, and the main directions have focused on the improvement of different processes, different formulations and attempts of various additives (such as curing agents, modifiers, formaldehyde capture agent) [3,4,16,24]. Among the various methods under consideration, the development of UF resins with lower F/U is the focus; this is because the resins with lower F/U have lower cross-linking during curing and can meet the requirements of use, which is also considered to be its unique advantage. Although many studies have been done on the morphology, crystal characteristics and structure analysis of UF resin [7,10,13,17,22]. So far, however, there are few studies on the changes in the morphology, crystallinity, and chemical structure of UF resin during the curing process. To clarify these changes will also be helpful to the formulation synthesis of UF resin and the analysis of curing mechanism. In order to achieve this goal, in this article, we used the traditional “alkali-acid-alkali” method to synthesize stable high F/U (1.4) and low F/U (1.0) UF resins, and passed polarizing microscope (POM), scanning electron microscope (SEM), X-ray diffraction (XRD), transmission electron microscope (TEM) and Fourier transform infrared spectroscopy (FT-IR) characterize the crystallinity, morphology and structure changes of UF resin before and after curing, and also explore the effects of different kinds of curing agents and their adding amount on UF resin were also studied.

## 2. Experiment

### 2.1. Material

Industrial grade urea (99%) and formaldehyde solution (37%) used to synthesize UF resin were purchased from Xilong Chemical Co., Ltd. (Shantou, China). The sodium hydroxide and formic acid used to adjust the pH during the synthesis of UF resin were purchased from Tianjin Damao Chemical Reagent Factory (Tianjin, China). Ammonium chloride (NH_4_Cl) and aluminum chloride (AlCl_3_) were used as curing agents for UF resin, and were purchased from Tianjin Kemeou Chemical Reagent Co., Ltd. (Tianjin, China, respectively mixed with 25% aqueous solution). Based on the non-volatile solid content of the resin, different proportions (1%, 3%, 8%) of curing agent are added to the synthetic resin. Analytical grade monomethylolurea (98%) and N-N’ dimethylolurea (98%) were purchased from Shanghai Maclean Biochemical Technology Co., Ltd. (Shanghai, China). Deionized water is obtained by ion exchange.

### 2.2. Experiment Methods

#### 2.2.1. Preparation of UF Resin

The pH value of formaldehyde was adjusted to 8.0–8.5, added to four-necked flasks, heated to 45 °C, added U_1_, heated to 90–92 °C in 25–30 min, and kept 40 min. Then, cool to 86–87 °C, adjust the pH value to 4.6–4.8, and heat up to 90–92 °C to continue the reaction. When the viscosity reaches 14–15 s (viscosity flow cup din 4, the following is the same), the pH value is adjusted to 5.8–6.0, and U_2_ is added to react. When the viscosity reaches 17–18 s, the pH value is adjusted to 6.0–6.2, the temperature is reduced to 88–90 °C, and then U_3_ is added for the reaction. When the viscosity reaches 20–21 s, the pH value is adjusted to 7.5–8.0, the temperature is reduced to 80 °C, the remaining U_4_ is added, and cooled to 50 °C, and the pH value is adjusted to 8.0–8.5. Finally, the obtained resins were cooled to room temperature, and the UF resins with molar ratios F/U of 1.4, 1.0 were prepared.

#### 2.2.2. Performance of UF Resin

The pH value of the resin was tested at 20 °C with a Lei Magnetic PHS-25 acidity meter (INESA Scientific Instrument Co., Ltd., Shanghai, China). The initial viscosity of the resin is measured by the NDJ-1 viscometer (Hengping Scientific Instrument Co., Ltd., Shanghai, China), using No. 2 rotor and rotating at 60 rpm. All samples were measured in triplicate and the average value was determined.

Pour about 1 g of UF resin into a disposable aluminum pan, and then dry it in a convection oven at 120 °C for 3 h. Determine the non-volatile solid content of UF resin by measuring the quality of UF resin before and after curing. All samples were tested in parallel for three times, and then the average value was taken as the result.

The curing time of UF resin is measured under boiling water conditions (the amount of curing agent added is based on 1% of the non-volatile solid content of the resin, and the curing agent is 25 wt% NH_4_Cl solution). All samples were measured in triplicate and the average value was determined. Table 1 summarizes all the measured results.

Free formaldehyde in the prepared UF resin was determined by sodium sulfite method according to the Reference [17]. All the measurements were done with three replications for each sample.

#### 2.2.3. Polarizing Optical Microscope (POM)

Monitor the microscopic image of the pure or hardener-added UF resin sample through a CCD camera (Ommicro, XPL-3230), and collect it through a computer equipped with a video capture card. The sample preparation was carried out by diluting the UF resin to a solid content of 1–2% with distilled water, sonicating it for 2 min, and then spreading the resin sample in a film on the glass.

#### 2.2.4. X-Ray Diffraction (XRD)

Two curing agents (NH_4_Cl, AlCl_3_) are added to UF with molar ratios of 1.4 and 1.0. The addition amount of each curing agent is based on the non-volatile solid content of the resin, which is 1%, 3%, and 8% respectively; Subsequently, the resin was cured at 120 °C for 60 min; the sample without curing agent was cured at 120 °C for 8 h to remove the moisture in the resin; the uncured UF sample was prepared by drying at 60 °C for 24 h. Then each type of resin is crushed into 200 mesh powder. The powder samples were tested at room temperature with an X-ray diffractometer (SMART type, Rigaku Co., Ltd.,Tokyo, Japan) Cukα-1 X-ray source (wavelength: 0.15406 nm); the scanning angle was 2θ, and the scanning range was 10°–60°, scanning step frequency is 0.02 °/min.

By applying the least square fitting program described by Hindeleh [29] and Park [7], the XRD spectrum is deconvolved to extract individual crystalline peaks and amorphous peaks to determine different molar ratios F/U The percentage of crystallinity of UF resin.

#### 2.2.5. Transmission Electron Microscope (TEM)

Using TEM (JEM2100F, Tokyo, Japan), the prepared cured UF resin powder was subjected to high-resolution image observation under an acceleration voltage of 75 kV, and selected area electron diffraction (SAED) was performed on the sample under an acceleration voltage of 150 kV. The sample preparation is that the solidified UF resin powder is prepared into 1wt% solution with distilled water and treated with ultrasonic for 2 min; the diluted sample is dripped onto the carbon coated film of 400 mesh copper mesh and dried, then the film on the copper mesh is dyed with 1 wt% uranyl acetate solution and dried again.

#### 2.2.6. Scanning Electron Microscope (SEM)

SEM (EVO18, Zeiss, Oberkochen, Germany) was used to study the surface morphology of pure UF resin films prepared with or without curing agent. The resin film was prepared by the method of forming the film on glass slides such as Park [28] (the treatment temperature and time before and after curing were the same as those in Section 2.2.4), then the surface of the film was spattered with gold, and then photographed. SEM observation was carried out under the accelerated voltage of 20 kV. Through the image analysis software, the spherical particle diameter of the cured UF resin was measured, the size of 20 particles of each sample was measured, and the average value was given.

#### 2.2.7. Fourier Infrared Spectrometer (FT-IR)

Crush each type of UF resin into 200 mesh powder, mix and grind KBr and the powder uniformly at a ratio of 1:100, weigh a certain amount of sample, and then put the white transparent tablet into the FT-IR instrument (NicoletiS50, Thermo Fisher, Madison, WI, USA) and set a blank KBr as the scanning background. The scanning resolution is 4 cm^−1^, the wavenumber range is 4000 to 500 cm^−1^, and the number of scanning accumulations is 32 times.

## 3. Results and Discussion

### 3.1. Properties of UF Resin with Different Mole Ratios F/U

Table 1 summarizes the properties of the prepared urea–formaldehyde resin. When the molar ratio decreased from 1.4 to 1.0, the solid content of the resin increased by 3.6%, and the apparent viscosity increased slightly. This may be due to the high content of formaldehyde in the high molar ratio urea–formaldehyde resin, and the solvation of formaldehyde increases the solubility of each component in the resin, resulting in low viscosity [18]. At the same time, the content of free formaldehyde determines the reaction activity of the resin [10]. As expected, the curing time of high molar ratio resin is shorter than that of low molar ratio resin. This is confirmed by the measurement results of curing time and free formaldehyde content in Table 1.

### 3.2. Crystalline Changes of UF Resin with Different Molar Ratios F/U

#### 3.2.1. Crystalline Morphology of UF Resin

**Figure 1:**
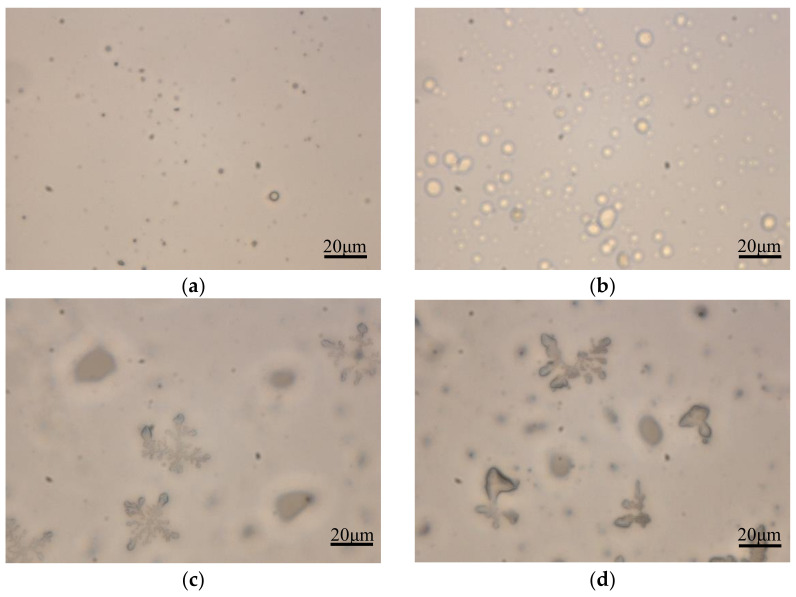
Polarized optical microscope photos of pure UF resin and UF resin with curing agent. (**a**) F/U = 1.4, (**b**) F/U = 1.0, (**c**,**d**) the effect of curing agent NH_4_Cl added 1 h on UF resin.

Figure 1 shows the POM photos of the pure resin and the resin after adding NH_4_Cl. Figure 1a,b is two different UF resins with transparent appearance. It can be noted that there are more spherical colloidal particles in the UF resin with a molar ratio of 1.0. At the same time, the spherical particles also exist in the UF resin with a molar ratio of 1.4, but the quantity and size are small. This is essentially consistent with the observation of Park [32] et al. for UF resins with different molar ratios. The formation and subsequent aggregation of colloidal particles have been shown to be the normal aging mode of amino resin. Aging or further promotion of the resin by other means (such as longer condensation time, addition of curing agent) can lead to whitening of the resin [33]. Figure 1c,d shows the change of the UF resin with NH_4_Cl, and the appearance of the UF resin solution begins to become turbid. Unlike spherical aggregates that produce flocculation in UF resin with storage time, some aggregates in Figure 1c,d show snowflake-like or dendritic structure. This may be because NH_4_^+^ is related to the reaction of free formaldehyde in the solution, the protonated formaldehyde in the solution decreases, the pH value in the solution decreases, the aggregation between colloidal particles is accelerated, and the cross-linking reaction between linear oligomers is intensified, which leads to the increase of aggregates in the solution. The formation of snowflake or dendritic aggregates in the solution may be tied to the formation of more crystal domain induced by the curing agent in the solution. Some studies have shown that spherical particles not only participate in the main mechanism of crystal growth, but also act as nucleation sites in the crystallization process; there are more ordered microcrystals in UF resin with low molar ratio, which may be related to the linear array of nano-substructure, which represents the aggregation of ordered molecules in UF resin [7,14].

#### 3.2.2. Crystalline Regions of UF Resin

**Figure 2:**
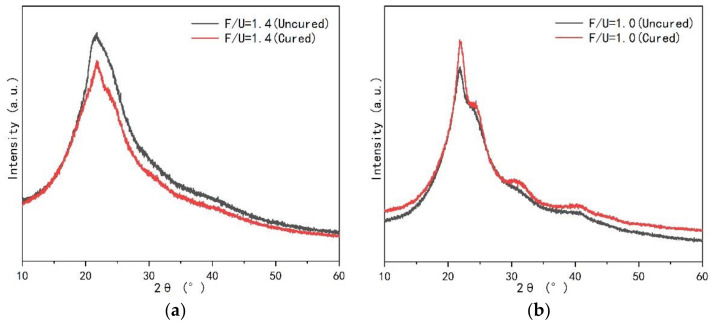
X-ray diffraction (XRD) patterns of UF resin with different molar ratios (F/U) before and after curing. (**a**) F/U = 1.4, (**b**) F/U = 1.0.

Figure 2 displays the comparison of XRD spectra of UF resins with molar ratios of 1.4 and 1.0 before and after curing. Figure 2a is the XRD spectrum of UF resin with F/U of 1.4 before and after curing. There is only one main peak at 2θ of 21.96°, and the main peak of the resin with F/U of 1.4 is slightly weakened after curing. Through calculation, it is found that the crystallinity after solidification is reduced from 29.02% to 28.30%. In contrast, the resin with F/U of 1.0 has a sharp main peak at 2θ of 21.96°, and an additional peak at 2θ of 24.64°, 31.26°, and 40.72°. When the resin is cured, the main peak in the XRD spectrum becomes sharp, the additional peak becomes obvious, and the crystallinity increases from 35.40% to 39.20% after curing, as showed in Figure 2b. These results indicate that the UF resin with higher F/U (1.4) has more amorphous regions; while UF resin with low F/U (1.0) has additional crystalline regions, showing more crystal structure. According to reports [25], the extra crystalline peaks in the XRD spectrum of the low molar ratio UF resin may be related to the spherical structure in the resin. At the same time, the crystallization peak position of UF resin with high F/U (1.4) and low F/U (1.0) and the additional peak position of UF resin with low F/U (1.0) did not change before and after curing.

The difference of crystallinity of UF resin with high F/U (1.4) and low F/U (1.0) before and after curing. This is primarily due to the decrease of linear methylene structure and the increase of branched chain methylene structure in UF with the increase of molar ratio [34]. The same molecule can participate in the crystalline or amorphous region of the semicrystal skeleton through different parts of its chain, resulting in no cross-linking of the-CH_2_- bridge in the crystalline region, but the hydrogen bonding force keeps the molecule in the crystalline state [13,25]. UF resin with low F/U contains more linear molecules forming the crystal region by hydrogen bond, while UF resin with high F/U contains more branched chain structure, which will help UF resins produce more cross-linked structures during the curing process, thereby reducing the crystalline area.

**Figure 3:**
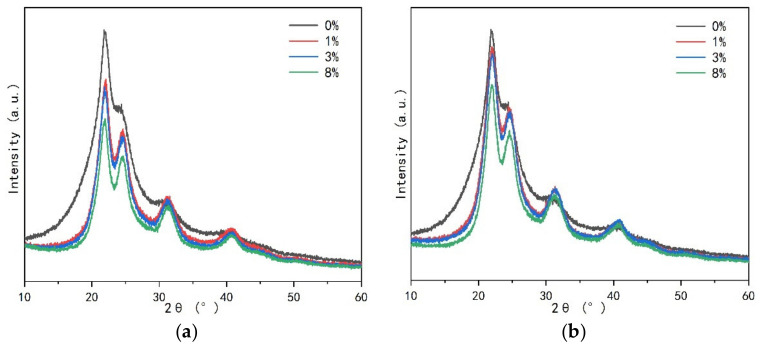
XRD pattern of UF resin with F/U = 1.0 under the influence of different content of curing agent. (**a**) NH_4_Cl, (**b**) AlCl_3_.

**Figure 4:**
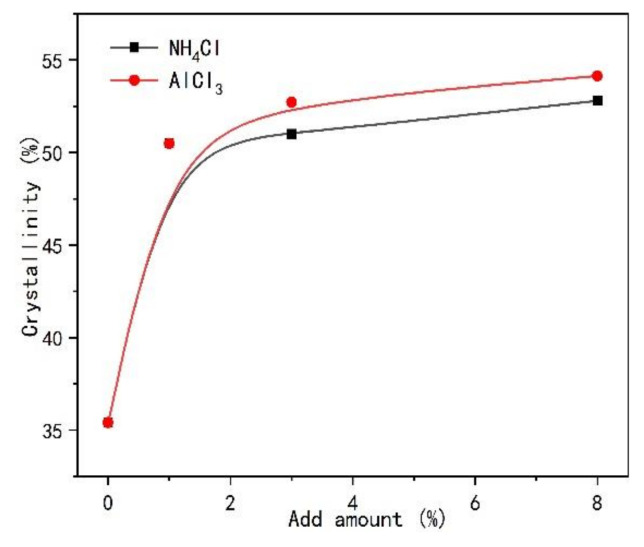
Comparison of crystallinity of UF resin with F/U = 1.0 under the influence of different types of curing agents and addition amounts.

In order to know whether the cured UF resin is inherent in the crystallization region. Different types and amounts of curing agents NH_4_Cl and AlCl_3_ were added to the UF resin with low F/U (1.0) to explore the changes of the crystallization zone, as showed in Figure 3. It can be seen that compared with Figure 2b, the introduction of the two curing agents significantly changed the shape of the XRD spectrum, and showed a very similar trend, and each peak shape became sharper, indicating that the curing agent obviously affected the strength of the crystal region. At the same time, it can be found that the position of each peak does not change with the type and amount of curing agent, indicating that the crystallization region of UF resin is inherent. Figure 4 shows the effect of different additional amount of two kinds of curing agent on the crystallinity of UF resin. It can be noted that the addition of curing agent significantly increases the crystallinity of UF resin. When the addition amount of curing agent increases to a certain extent, the crystallinity no longer increases dramatically. Studies have shown that when the acid content in UF resin is too high, such as pH below 4.0, it may lead to the degradation of linear oligomer chain containing cured UF resin, which will lead to the decrease of resin crystallinity [15]. Therefore, the crystallinity of the resin with AlCl_3_ is slightly higher than that of the resin with NH_4_Cl, which may be due to the stronger acidity of AlCl_3_ in the resin, which leads to the increase of the crystallization region of the resin, which further affects the crystallinity.

**Figure 5:**
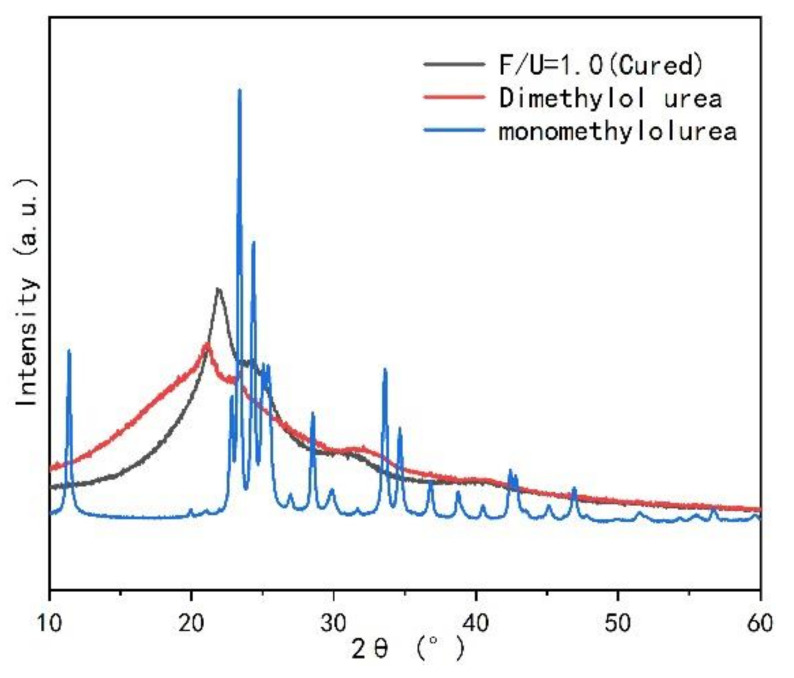
Comparison of XRD patterns of monomethylol urea and dimethylol urea with cured UF resin.

In order to analyze the crystal region of UF resin in detail, monomethylol urea and dimethylol urea with purity of 98% were selected and compared with cured low F/U (1.0) UF resin for XRD diffraction patterns, as shown in Figure 5. It can be seen from Figure 5 that there is a great difference in XRD spectra between monomethylol urea and cured UF resin, and the peaks in the crystallization region of cured UF resin do not overlap with those of monomethylol urea. These results indicate that the crystal structure of monomethylol urea does not contribute much to the cured UF resin. The XRD spectra of dimethylol urea and the cured UF resin (1.0) are very similar, which indicates that the crystallization region of UF resin with low F/U (1.0) is likely to be related to dimethylol urea.

#### 3.2.3. Crystal Characteristics of UF Resin

**Figure 6:**
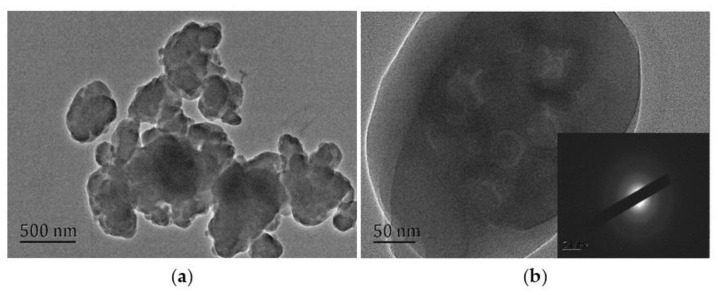
Transmission electron microscope (TEM) and selected area electron diffraction (SAED) images of curing UF resin (F/U = 1.0, 3% NH_4_Cl). (**a**) TEM images of resin particles, (**b**) ED patterns of resin particles.

Figure 6 shows the TEM and SAED diagrams of cured UF resin. As shown in Figure 6b, through the selective area electron diffraction operation on the resin sample, the ED mode shows multiple concentric rings (roughly three clear rings), indicating that the cured UF resin (1.0) has an isotropic polycrystalline structure and has no obvious preferred orientation relative to the incident electron beam. The radius (R) and the crystal plane spacing (d) of concentric rings are determined by the camera constant (K). Many studies have shown that [7,17,25], cured UF resin has the characteristics of cubic unit cells. Through the measurement of the SAED diagram in the lower right corner of Figure 6b, it is found that the square R^2^ of the radius of each ring is satisfied, R_1_^2^: R_2_^2^: R_3_^2^ = 1:1.96:2.96 (unnatural number) = 2:4:6, which accords with the extinction law of the body-centered cubic lattice, so the crystal plane index (h k l) marked from inside to outside is as follows: (110) (200) (211). At the same time, according to the CCD camera source image taken by TEM, the distance between the first ring and the center is obtained using software analysis and measurement, and the crystal plane spacing d is calculated to be 2.90 nm. Depending on formula (1), the side length a of the unit cell is 4.08 nm.

(1) $$d=\frac{a}{\sqrt{h^{2}+k^{2}+l^{2}}}$$

### 3.3. Morphological Changes of UF Resins with Different Molar Ratios F/U

**Figure 7:**
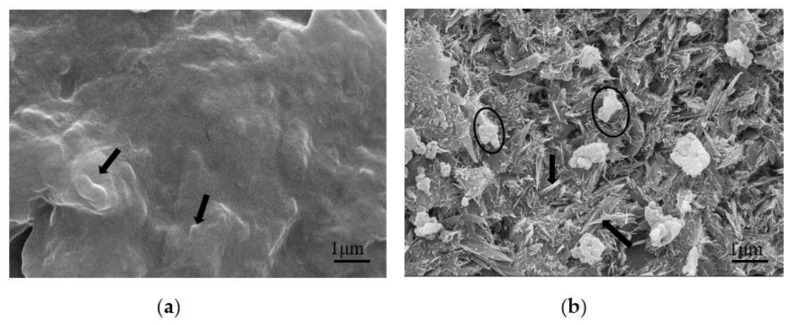
Scanning electron microscope (SEM) comparison of uncured UF resin with different molar ratios. (**a**) F/U = 1.4, there are surface protrusions (arrows) of spherical particles in the section. (**b**) F/U = 1.0, there is an aggregation of spherical particles (ellipse) in the section. The crystal aggregate is composed of a series of crystals of different shapes. The crystals have the form of flat plates with sharp ends on the edges, which can be identified as single crystals (arrows).

Figure 7 shows the SEM comparison of the cross-sections of uncured UF resins with molar ratios of F/U of 1.4 and 1.0. It can be seen from the cross-section of the film that the resin with F/U of 1.4 shows an uneven shape, and the spherical nodular surface protrusions in a part of the area are clearly visible, as showed in Figure 7a. However, in Figure 7b, there are many shaped spherical particles, aggregates, and flat plate-like crystals. Flat plate crystals are considered to represent the progressive or complete growth form of crystals. On the surface, some crystals are needle-like, but careful observation shows that the needle-like appearance comes from the peculiar direction of the crystal, in which only the edge of the crystal can be seen. The morphological difference between the two uncured resins may be related to the more complex cross-linked network structure of the UF resin with F/U of 1.4 and the solvation of formaldehyde, which affects the existence of spherical particles and the development of crystal domains [13,25].

**Figure 8:**
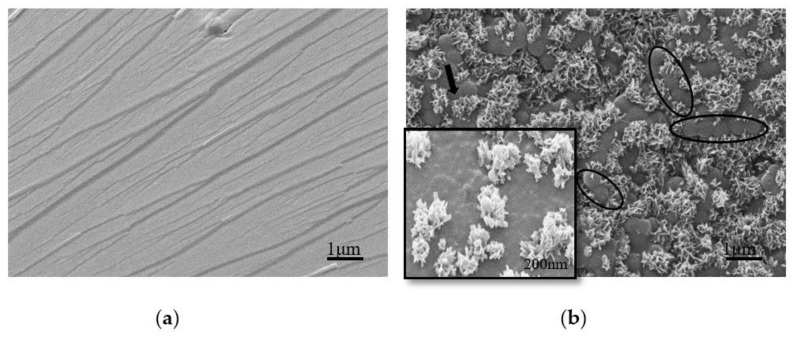
SEM comparison of cured UF resins (no curing agent added) with different molar ratios. (**a**) F/U = 1.4, (**b**) F/U = 1.0, a large number of spherical particles appeared on the section, and some spherical particles were clustered in the form of beads (ellipse), surrounded by many snowflake-shaped primary particles (arrows) composed of flat plates and filamentous crystals. Illustration: High magnification view at the arrow in the upper left corner of the photomicrograph.

Figure 8 shows the SEM comparison of cured UF resins with molar ratios of 1.4 and 1.0. In the cross section of the cured resin, the resin with F/U of 1.4 did not show any specific morphology, which may be due to the hydrolysis reaction caused by the water generated by the high molar ratio UF resin cured at high temperature; the small holes on the surface are caused by caused by evaporation of moisture and formaldehyde [21]. However, the morphology of UF resin with low molar ratio is completely opposite, the appearance of a large number of spherical particles and the linear arrangement of some spherical particles in clusters, which is considered to be the source of a greater degree of crystal domain; at the same time, the particle surface attached a lot of snowflake-like primary particles, which may be cured at high temperature, flat and filamentous crystals developed to a more advanced degree. The spherical properties of the crystal domain of UF resin have been reported by small angle X-ray diffraction [7]. In other words, elevated temperature curing has an obvious effect on the crystal of UF resin with low molar ratio, and the appearance of a large number of spherical structures on the fracture surface may be related to the change of crystal domain.

**Figure 9:**
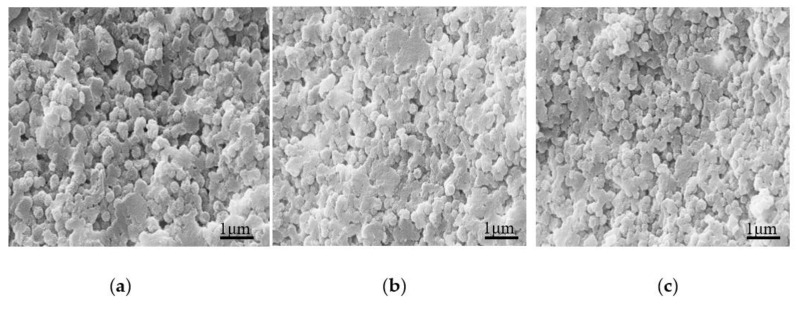
SEM comparison of UF resin with a molar ratio of 1.0 cured under different NH_4_Cl additions. (**a**) 1%, (**b**) 3%, (**c**) 8%.

**Figure 10:**
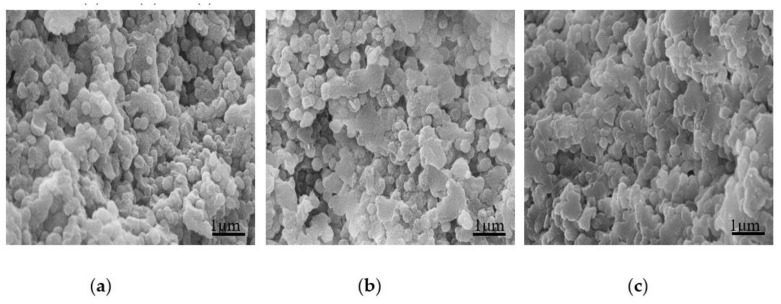
SEM comparison of UF resin with a molar ratio of 1.0 cured under different AlCl_3_ additions. (**a**) 1%, (**b**) 3%, (**c**) 8%.

In order to understand the morphology of cured UF resin in detail. We added different amounts of NH_4_Cl and AlCl_3_ to the UF resin with a molar ratio of 1.0 to explore the change of the cured resin, as shown in Figure 9 and Figure 10. Compared with Figure 8b, the morphology of the cured resin is quite different with the addition of curing agent; when the curing agent is added, the snowflake-like primary particles composed of flat crystals around the spherical particles in Figure 8b disappear, indicating that the curing agent has an obvious effect on the morphology of UF resin; Figure 9 and Figure 10 appear a large number of spherical particles, which may be that the curing agent promotes the development of these primary particles to a more advanced level. In other words, the appearance of a large number of resin spherical particles under the curing agent is closely related to the sharp increase in the crystallinity of the resin under the curing agent analyzed in Section 3.2.2.

**Figure 11:**
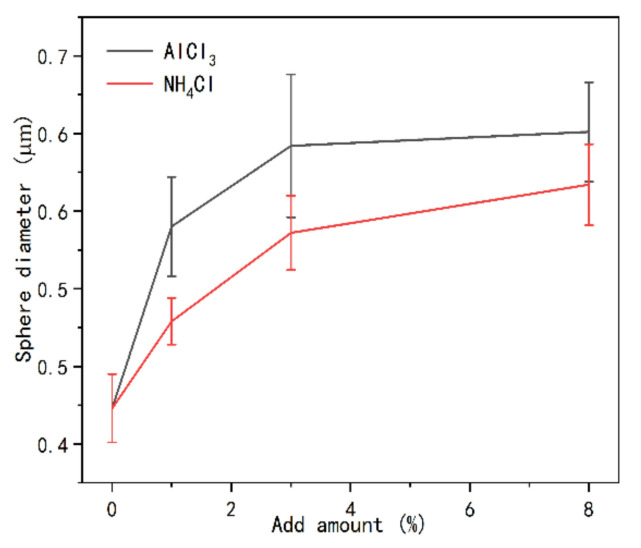
Comparison of the sphere diameters of UF resin with a molar ratio of 1.0 after curing under the influence of different addition amounts of NH_4_Cl and AlCl_3_.

The variation of the diameter of the spherical structure with the addition of curing agent in Figure 9 and Figure 10 was measured by image analysis software, as shown in Figure 11. The introduction of two kinds of curing agents led to the increase of the diameter of spherical particles in the cured resin; at the same time, the particle diameter of the resin with AlCl_3_ was slightly higher than that of the resin with NH_4_Cl, and the diameter of spherical particles basically reached the maximum when the amount of curing agent reached 3%. Then, with the increase of the amount of curing agent, the diameter of spherical particles remained stable. This may be due to the strong acidic environment caused by both curing agents in the resin. With the increase of curing agent content, the acidic condition of UF resin during curing is stronger, thus providing a greater cross-linking density [35]. As reported by Dunker [20], due to the presence of double-layer protonated formaldehyde, a colloidal structure was formed in the UF resin. Ferra [36] et al. found that the resin curing process is often accompanied by the consumption of urea and linear oligomers, resulting in the formation of more aggregates. Therefore, the increase of NH_4_Cl content in UF resin may destroy the double-layer protonated formaldehyde structure, form hexamethylenetetramine salt and strong acid, and promote the consumption of oligomers under acidic conditions, thus increasing the diameter of the spherical structure of UF resin. When AlCl_3_ is used as curing agent, the diameter of spherical particles is slightly larger than that of the resin with NH_4_Cl, which may lead to a stronger acidic environment in the resin system and aggravate the consumption of linear oligomers. At the same time, the high valence Al^3+^ will also destroy the double-layer protonated formaldehyde structure of the resin. However, the diameter of spherical particles remained stable after the addition of curing agent reached 3%, which was not only related to the amount of free formaldehyde in UF resin, but also related to the hydrolysis reaction of resin in strong acid environment, just like the analysis of crystallinity change in Section 3.2.2. It has been reported that the hydrolysis of cured UF resin can begin at the site of chloride on the surface of the spherical structure; with the increase of curing agent content, the amount of carbon and oxygen decreases, while the amount of nitrogen and chlorine increases as expected [35].

### 3.4. Structural Changes of UF Resins with Different Molar Ratios F/U

**Figure 12:**
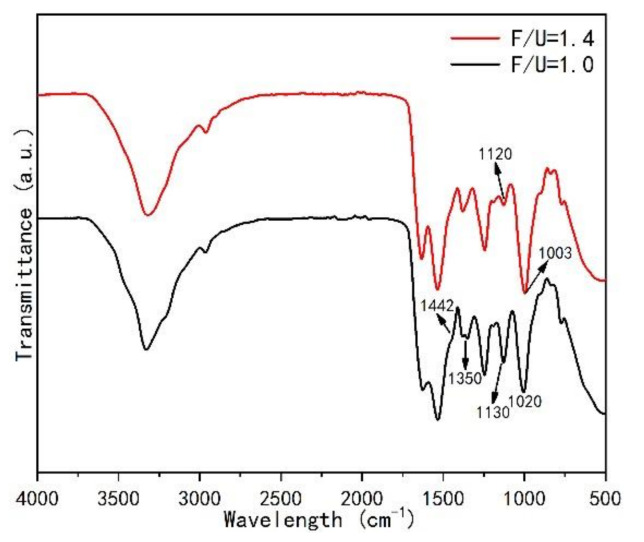
Comparison of Fourier infrared spectrometer analysis (FT-IR) spectra of UF resins with molar ratios of 1.4 and 1.0.

Figure 12 shows in the FT-IR spectra of two mole ratios of UF resins. It can be found that the spectra of UF resins with F/U 1.4 and 1.0 are very similar to each other, but there are some differences in specific chemical bands, as showed by the arrow marks in Figure 12. The spectrum of the resin with F/U of 1.0 showed more absorption bands than the resin with F/U of 1.4. The bands of 1442, 1350 and 1020 cm^−1^ were found in the resin with F/U of 1.0, which was not found in resin with F/U of 1.4. The bands at 1442 and 1350 cm^−1^ are attributed to C-H bending vibration and the bending vibration of the amide II band, respectively. The band at 1130 cm^−1^ is related to the C-O-C associated with monomethylol urea, which indicates that there is monomethylol urea exists in the UF resin with F/U of 1.0 [13,37]. At the same time, the band at 1003 cm^−1^ in the spectrum of the UF resin with F/U of 1.4 corresponds to the C-O in dimethylol urea [13], indicating that there are various dimethylol urea in resin with F/U of 1.4. These results indicate that high F/U resins contain more branched structures, while low F/U resins are mainly composed of linear structure segments, such as monomethylol urea and ether bonds. The difference between these structures may be related to crystallinity and morphology, that is, more linear segments are conducive to the formation of more crystal regions and the appearance of spherical particles.

**Figure 13:**
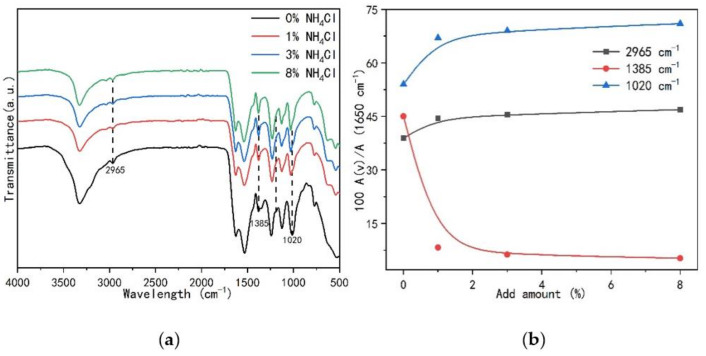
Comparison of FT-IR spectra of UF resin with a molar ratio of 1.0 under different curing agent content. (**a**) Three absorption bands (dashed lines) used for quantitative analysis in FT-IR; (**b**) the three absorption bands are based on the quantitative results of the carbonyl group at 1650 cm^−1^.

Figure 13 shows the FT-IR spectrum of the UF resin with F/U 1.0 under the influence of different amounts of curing agent. At the same time, the main bands were normalized based on the carbonyl group at 1650 cm^−1^ for quantitative comparison. The band at the position of 2965 cm^−1^ in the spectrum is attributed to the C-H stretching vibration, which may be the result of the joint contribution of dimethylene ether bond (-CH_2_-O-CH_2_-), hydroxymethyl (-CH_2_OH) and methylene (-N-CH_2_). The band at 1385 cm^−1^ position is related to the contribution of -CH_2_OH, and the band at 1020 cm^−1^ is ascribed to the methylene bridge (-N-CH_2_-N-) [37]. However, the peak height of the carbonyl group at 1650 cm^−1^ does not change with the content of the curing agent, so it can be used as a reference peak to normalize the absorbance of each band with the absorbance of the carbonyl group, and compare each band quantitatively, as shown in Figure 13b. The results of quantitative comparison showed that with the increase of the amount of curing agent, the content of methylene bridge increased, while the content of hydroxymethyl decreased to a lower level. This is mainly because the acidity increases with the increase of the content of curing agent, which leads to the acceleration of the reaction, which is consistent with the condensation principle of UF resin, that is, methylene bridge bonds are formed by dehydration condensation between hydroxymethyl groups, and water molecules are formed. When the cross-linking structure is formed, the content of each bond will change accordingly.

### 3.5. Crystallization Model of Urea–Formaldehyde Resin during Curing Process

The crystallization of UF resin exists in both the synthesis process and the curing process [15,22]. Several previous studies have reported the role of linear structural segments in the formation of crystals in UF resins [19,20,26]. At the same time, it combines the characterization and analysis results of UF resin before and after curing. It can be seen that there are spherical colloidal particles in both resins. Spherical colloidal particles are closely related to the development of crystals. The difference between high F/U (1.4) and low F/U (1.0) UF resins lies in that the former has more branched chain structure and solvation caused by free formaldehyde and hydrophilic groups. Finally, it leads to the difference of morphology and crystallinity in the curing process.

**Figure 14:**
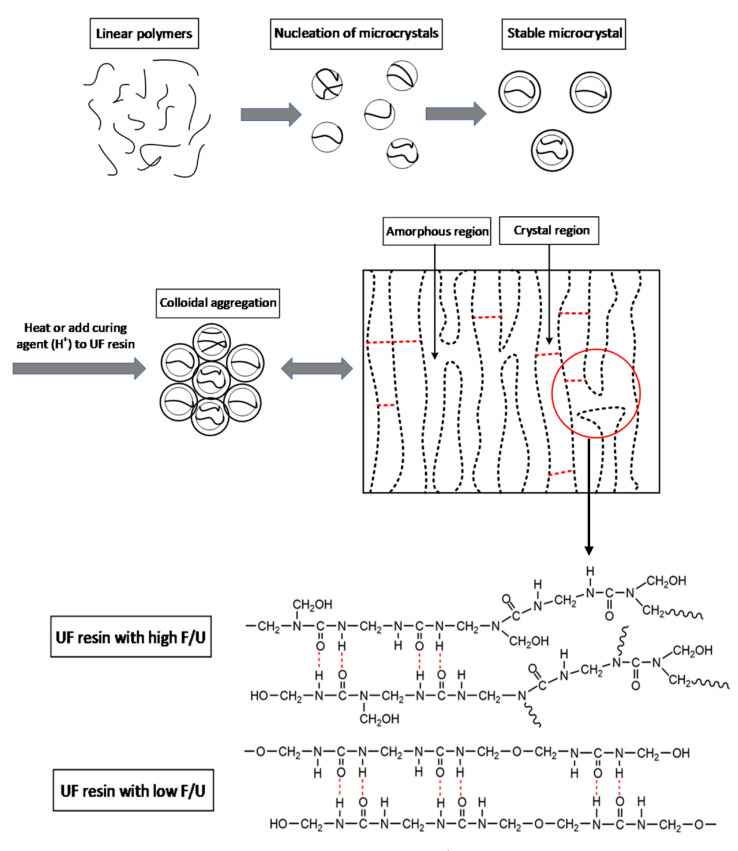
Possible growth methods of UF resin crystals or spherical particles.

As showed in Figure 14, the growth model of crystals or spherical particles in UF resin is shown. In the synthesis process of urea–formaldehyde resin, the primary microcrystalline particles were formed in the linear molecular chain due to the decrease of solubility in the alkaline stage, and then a stable microcrystalline structure began to form with the adjustment of pH value and the addition of urea [19,22,26]. When the UF resin is cured by heating or adding a curing agent (H^+^), the colloidal particles aggregate due to the decrease in stability. At the same time, heating or adding curing agent provides a greater driving force for crystal growth. The crystal growth of UF resin is inevitably related to the branched chain structure. During the curing process, the higher branched chain structure in UF resin does not contribute to the development of crystal region. Because under the condition of high temperature or curing agent (H^+^), the hydrophilic groups on the branched structure will form a cross-linked network structure and are more prone to hydrolysis [17], which will reduce the hydrogen bonds responsible for crystallization, thus reducing the crystallinity of UF resin.

**Figure 15:**
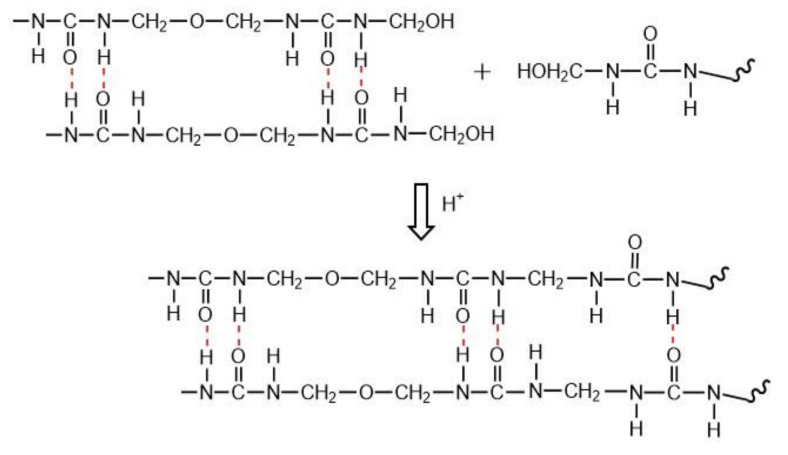
Growth of Crystalline region of UF resin with low molar ratio F/U in the presence of the curing agent (H^+^).

Figure 15 shows the growth of the crystalline region of low molar ratio urea–formaldehyde resin in the presence of curing agent (H^+^). The results of FT-IR quantitative analysis showed that with the increase of curing agent content, methylene content increased and hydroxymethyl content decreased to a very low level. Finally, it leads to the obvious increase of crystallinity.

## 4. Conclusions

This paper reports the relationship between crystallinity, morphology and structure of urea–formaldehyde resin with different molar ratio before and after curing, which is helpful to better study the curing process of urea–formaldehyde resin. Understanding these changes will also be helpful to the formulation synthesis and curing mechanism analysis of urea–formaldehyde resin.

(1) The UF resin with high F/U (1.4) showed amorphous, while the resin with low F/U (1.0) showed crystallization region. The common crystallization peak and the position of additional peak did not change before and after curing, and at the same time, it did not change with the change of curing agent type and addition.
(2) The cured UF resin with a low F/U (1.0) has the characteristics of polycrystal and body-centered cubic cell.
(3) The change of crystallinity may be related to the appearance of spherical particles, which may be developed from flat crystals to a more advanced level, and the existence of curing agent significantly promotes the development of crystal region. Spherical particles are part of the crystal, or the nucleation site of the primary crystal.
(4) The branched chain structure of UF resin with high F/U may affect the curing presentation mode of the resin, such as crystallinity, morphology and so on. The linear segment may affect the orderly development of the crystal region or the packing of spherical particles.

## Figures and Tables

**Table 1:** Properties of urea–formaldehyde (UF) resins of two different mole ratios F/U.

F/U Mole Ratio	Solid Content (%)	Viscosity (mPa.s)	Curing Time (s)	Free Formaldehyde (%)
1.4	52.0	42	81	0.45
1.0	55.6	48	122	0.24

## Data Availability

The data presented in this study are available on request from the corresponding author.
